# So Much Moulage, So Little Time: A Guide to Performing Moulage for Mass Casualty Scenarios

**DOI:** 10.7759/cureus.18780

**Published:** 2021-10-14

**Authors:** Josh Brooks, Asit Misra, Brad D Gable

**Affiliations:** 1 Graduate Medical Education, OhioHealth Doctors Hospital, Columbus, USA; 2 Surgery (Division of Emergency Medicine) and The Gordon Center for Simulation & Innovation in Medical Education, University of Miami Miller School of Medicine, Miami, USA; 3 Emergency Medicine, Riverside Methodist Hospital, Columbus, USA

**Keywords:** simulation, moulage, mass casualty, multi-victim, disaster, standardized patients, low-cost task trainers, experiential learning, simulation realism, process & performance improvement

## Abstract

Simulation has become a central component of healthcare education. Allowing learners to experience low-frequency high-risk situations, such as a mass casualty event, in a safe learning environment is a basic tenet of simulation-based education in healthcare. Creating realistic simulations often involves advanced moulage to accurately represent illness and injury. However, providing advanced moulage for mass casualty exercises can be time-consuming, resource-intensive, and costly. Here we discuss a novel means to execute moulage for multiple victims while maintaining a high level of realism. We executed two simultaneous mass casualty exercises as part of medical student education and employed our novel 3-step moulage process. Step 1-Preparation included case development, generation of a victim list, and victim designation into “zones” within the simulation. Step 2-Creation entailed making wounds, in-house 3D printing materials, and assembling each victim’s moulage bag. Step 3-Application was an assembly line method of executing all victims’ moulage on the day of the simulation. This method of moulage supported the highly realistic simulation activity that learners have come to expect while decreasing time, resources, and cost.

## Introduction

Simulation has become an integral component of education for healthcare professionals [1]. Allowing learners to experience low-frequency high-risk situations, such as a mass casualty event, in a safe learning environment is a basic tenet of simulation-based education in healthcare. One of the critical aspects of the simulated learning environment is engendering enough realism to enable learners to apply context to the clinical problem they are encountering [2].

Simulation-based mass casualty exercises (SBMCE) are conducted to train healthcare workers and students to prepare them for low-frequency incidents that require expertise in the management of multiple victims [3,4]. Live SBMCEs, as opposed to tabletop and computer-based exercises, use multiple victims (standardized patients [SP] and mannequins) to mimic an incident. Based on the learning objectives of these exercises, the success of delivering an optimal and realistic training environment depends on the expertise of the simulation faculty, subject matter experts, and the mastery of the simulation specialists [5]. One key element of realism is the moulage of the victims to replicate real-life pathology or injury [6]. Previous studies have demonstrated the use of moulage as an excellent tool to improve the behavioral outcomes of learners as they feel more immersed in the training environment and apply their skills as they would have applied them in a real-world setting [7]. This includes triaging multiple victims and prioritizing care for salvageable victims in a mass casualty training simulation. There are several different ways moulage can be utilized in a simulated scenario and it is a crucial element of healthcare simulation [8]. However, effective moulage can be time-consuming and pose a significant challenge in conducting large-scale exercises due to the limited time to apply moulage to the victims [4]. In response, we developed our innovative method for applying moulage for SMCEs. Our method supports educational strategies as part of curriculum development [9], as it helps faculty and educators achieve the psychomotor objectives of their curriculum for SBMCE by enhancing the realism of injuries on victims. Here we describe our simple 3-step process for executing moulage of victims (SPs and mannequins) in a mass casualty simulation.

## Technical report

Context

The SBMCE consisted of two different scenarios that were performed concomitantly and then repeated over two hours. Learners were divided into two groups of 20 each to participate in each scenario. The first scenario was a chemical blast, in a storage area of a barn, with organophosphate (OP) exposure having a mix of victims with blast injuries, OP exposure, or combinations thereof. The second scenario was a high-energy bomb blast and a resultant fire in a pub. The victims had a combination of blast injuries, thermal injuries, and smoke inhalation. In order to accomplish the learning objectives, the scenarios required a total of 29 SPs and six mannequins as victims. To overcome time constraints and limited manpower, a novel approach to the application of patient moulage was developed.

Input

Total number of victims-35. Total number of simulation technicians involved in SBMCE development-3. Total number of simulation technicians to apply moulage on day of event-5. Total time to create and package materials-40 hours. Total time for moulage application-1 hour. Total cost per victim-$6.75.

Material cost breakdown:

Wounds-Smooth-On, Inc. (PA, USA) products: DragonSkin™ (wound), EcoFlex™ (wound), Skin Tite™ (wound adhesive); DAP® Products Inc. (MD, USA) Plaster of Paris (wound mold)-$1.50

Shrapnel - Inland (CA, USA) PETG 3D printer filament-$1

Gallon-sized resealable bags-$.025

Thrift store clothing-$4

Process

**Figure 1:**
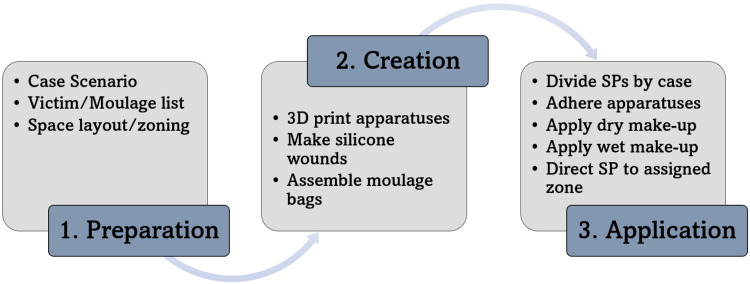
OhioHealth Simulation 3-Step Moulage Process SP: standardized patients

The execution of the OhioHealth Simulation 3-Step Moulage Process is shown in Figure 1. Step 1-Preparation: included case development, generation of a victim list, and victim designation into “zones” within the simulation field. Step 2-Creation: entailed making wounds, 3D printing materials, and assembling each victim’s moulage bag. Step 3-Application: utilized an assembly line method of executing all victims’ moulage on the day of the simulation.

Preparation began with the writing of the case scenarios that directed moulage development. Utilizing our institution’s scenario templates, we defined the location of the event, layout of the scenario, and educational objectives to categorize the number and types of victims required for each SBMCE. We then generated a victim list and assigned each victim a number, zone (physical location within the simulated field), medium (mannequin or standardized patient), triage category, mechanism and types of injuries commensurate with the triage category, and moulage required (wounds, symptoms, clothing) (Table 1). We also created a victim label that was worn on the clothing of each victim.

**Table 1:** Example of Blast/OP case victim list *Staging Zones designate the placement of the victim within the scenario field. In this example, Zone 1 to Zone 3 correlated with the distance from the blast epicenter. OP: organophosphate

Scenario Parameters	Moulage Details
Victim	#7
Staging zone*	2
Medium	Mannequin
Triage category	Red
Chief complaint/injury	Shrapnel in abdomen, difficulty breathing
Airway	No foreign body
Breathing	Breathing once every 3 seconds
Circulation	Color returns to fingertip in 4 seconds
Wounds/Injuries	Shrapnel impaled in abdomen, foaming at mouth, sweaty
Clothing	Shirt with a hole where shrapnel is protruding
Dry make-up	3D printed metal shrapnel set in silicone, silicone foam on mouth
Wet Make-up	Large amount of blood from an impaled wound
Victim label	Foaming at mouth, eyes watering, sweaty, breathing once every 3 seconds, color returns to fingertips in 4 seconds, responds to pain only

**Figure 2:**
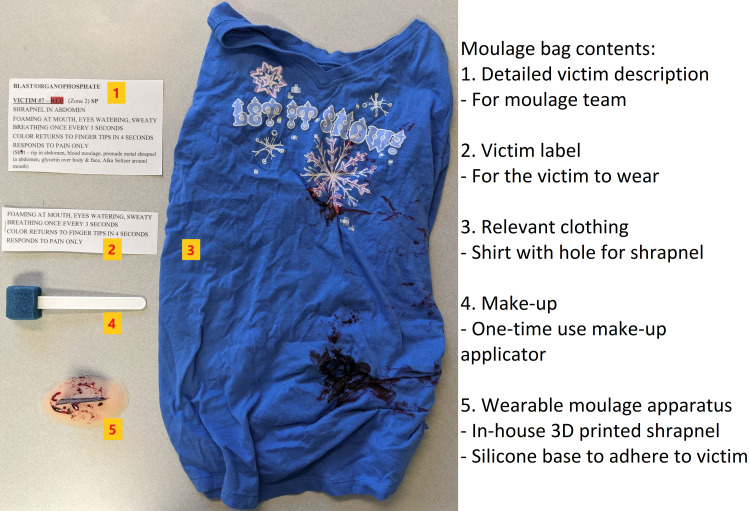
Moulage Bag Contents

Utilizing the victim lists above, we then created our innovative “moulage bag” (Figure 2) for each victim. The moulage bag contained all information and equipment necessary for the moulage of the victim, including details of all injuries for the simulation team, victim label, clothing, make-up, and pre-made wounds/burns necessary for the victim. Our simulation team utilized 3D printed elements (shrapnel, rebar, exposed bones), hand-made materials (lacerations, abrasions, burns), and purchased materials (evisceration, amputation). Each moulage bag was compiled in advance of the day of the mass casualty simulation training.

**Figure 3:**
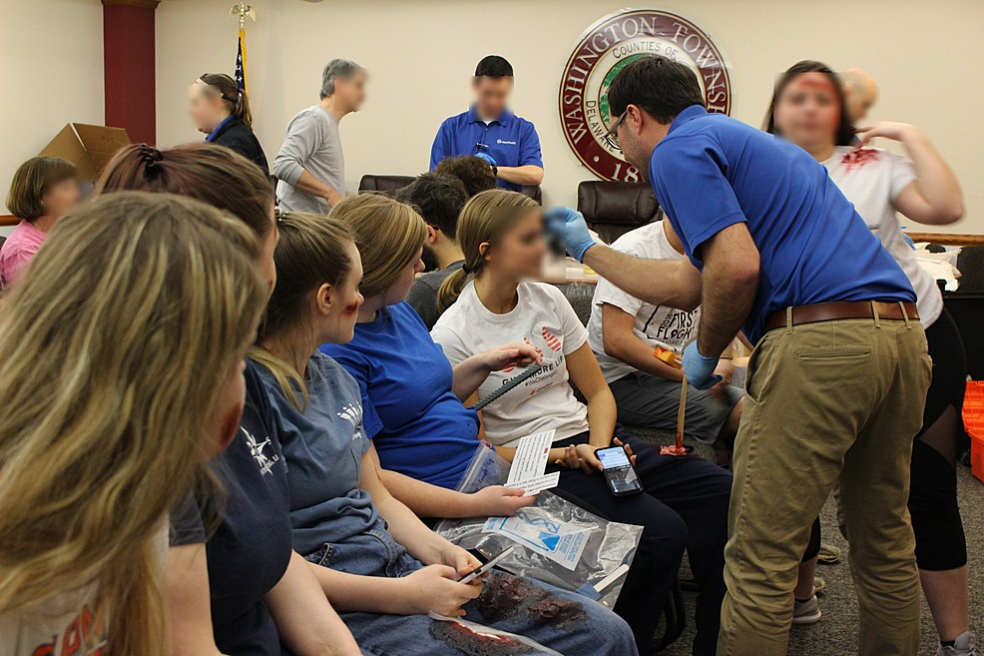
Moulage Assembly Line

**Figure 4:**
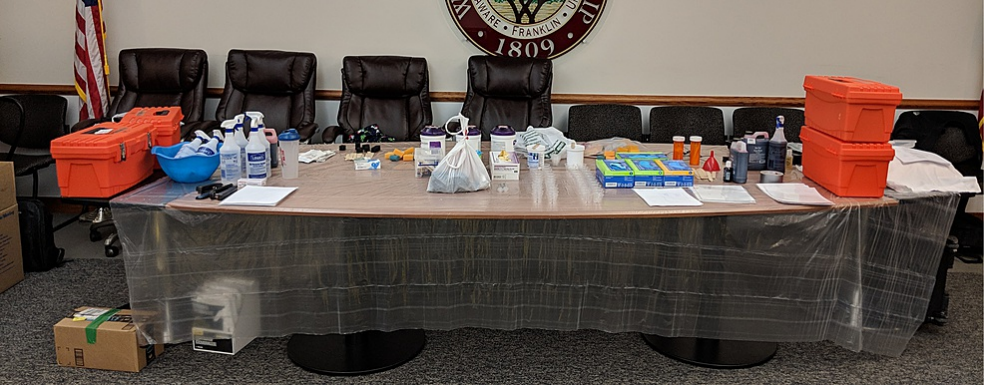
Draped Table

All moulage, regardless of the medium, was applied on the day of the mass casualty simulation training. To expedite the process for mannequin moulage, victims were placed in their appropriate zone, and their injury and dry make-up moulage elements were applied in place. Standardized patient moulage was performed in two “moulage assembly lines” (Figure 3). To do this, we draped the floor and furniture with waterproof sheeting to prevent any staining of the permanent fixtures (Figure 4). Each standardized patient was assigned a victim number for their scenario and given the appropriate moulage bag. Two rows of chairs, back-to-back, were designated to each scenario. When SPs were approached by a simulation technician, they presented them with their bag to begin the moulage process. The moulage process was divided into three rounds. Round 1 was the application of any required adhesives. Victims had their pre-made wounds applied using fixative agents. Round 2 entailed the application of moisture-free make-up to create redness, bruising, eschar, and some burns. Victims also applied their victim label at this time. Round 3 included the application of wet make-up, such as blood, body fluid, and OP-simulated materials. Finally, as all SPs were completing their moulage, they were briefed on their injuries, given instructions on how their injuries would present clinically, and reviewed safety information. Additionally, the mannequins underwent the application of their wet make-up at this time as well.

Lastly, SPs were staged by directing them to the designated zone of their respective scenario. A reset period, between scenarios, allowed for moulage adjustments and touch-up.

## Discussion

Our novel and reproducible 3-step moulage process for multiple victims in a mass casualty simulation exercise is beneficial for any simulation program or simulation-based training exercises. This process is especially beneficial to those programs that have limited simulation staff to support the activities planned. Our method of mass casualty moulage successfully increased the level of realism. Studies have reported that increased realism has been shown to improve the learners’ engagement and suspension of disbelief in simulated scenarios [10-12]. The key steps in our moulage process were preparation, creation, and application. There are several novel aspects to this process such as a comprehensive victim list, an individualized moulage bag, and an assembly line approach. All of these aspects allowed our team to quickly and efficiently apply moulage to all victims within the constraints previously mentioned. Additionally, time savings can be achieved during future SBMCEs since most materials can be reused.

The feedback from the simulation team was that this process was very systematic and made the application of moulage easier and faster than our previous methods. To that end, we required less of our simulation staff to be on-site the day of the education, as much of the moulage preparation was completed in advance. Rather than purchasing all materials, creating the wounds and props in-house allowed for additional cost savings. Additionally, we were able to incorporate more victims with increasingly realistic injuries into our scenarios as a result of this process.

## Conclusions

Our standardized 3-step moulage process can be applied to nearly any simulation-based training. This method is especially beneficial in those educational environments where simulation staff are limited or when moulage of multiple SPs or mannequins is required within a tight schedule. This process will allow for large-scale SBMCE to be conducted in a more realistic, faster, and more cost-effective manner in the future.

Recommendations for practice:

1. Moulage for SPs and mannequins in simulations should follow a 3-step process: Preparation, Creation, Application.

2. Pre-made moulage bags improve efficiency and should contain: victim description, victim label, relevant clothing, make-up, and wounds/burns.

3. An assembly line approach to moulage allows for fast and efficient application of wounds and make-up and should be conducted in a systematic fashion: adhesives, dry make-up, wet make-up.
